# Health system capacity in Sydney, Australia in the event of a biological attack with smallpox

**DOI:** 10.1371/journal.pone.0217704

**Published:** 2019-06-14

**Authors:** Chandini Raina MacIntyre, Valentina Costantino, Mohana Priya Kunasekaran

**Affiliations:** 1 Biosecurity Program, Kirby Institute, Faculty of Medicine, The University of New South Wales, Sydney, New South Wales, Australia; 2 College of Public Service and Community Solutions, Arizona State University, Tempe, Arizona, United States of America; The University of Hong Kong, CHINA

## Abstract

Planning for a re-emergent epidemic of smallpox requires surge capacity of space, resources and personnel within health systems. There are many uncertainties in such a scenario, including likelihood and size of an attack, speed of response and health system capacity. We used a model for smallpox transmission to determine requirements for hospital beds, contact tracing and health workers (HCWs) in Sydney, Australia, during a modelled epidemic of smallpox. Sensitivity analysis was done on attack size, speed of response and proportion of case isolation and contact tracing. We estimated 100638 clinical HCWs and 14595 public hospital beds in Sydney. Rapid response, case isolation and contact tracing are influential on epidemic size, with case isolation more influential than contact tracing. With 95% of cases isolated, outbreak control can be achieved within 100 days even with only 50% of contacts traced. However, if case isolation and contact tracing both fall to 50%, epidemic control is lost. With a smaller initial attack and a response commencing 20 days after the attack, health system impacts are modest. The requirement for hospital beds will vary from up to 4% to 100% of all available beds in best and worst case scenarios. If the response is delayed, or if the attack infects 10000 people, all available beds will be exceeded within 40 days, with corresponding surge requirements for clinical health care workers (HCWs). We estimated there are 330 public health workers in Sydney with up to 940,350 contacts to be traced. At least 3 million respirators will be needed for the first 100 days. To ensure adequate health system capacity, rapid response, high rates of case isolation, excellent contact tracing and vaccination, and protection of HCWs should be a priority. Surge capacity must be planned. Failures in any of these could cause health system failure, with inadequate beds, quarantine spaces, personnel, PPE and inability to manage other acute health conditions.

## Introduction

Smallpox is a category A bioterrorism agent, despite being declared eradicated in 1980[1]. The virus is retained in high security biosafety level 4 laboratories in the United States and Russia [2]. The variola genome is fully sequenced and could be synthesized in a laboratory [3]. This was previously thought to be unlikely, but Canadian researchers synthesized a closely related orthopox virus in 2017 and published the methods in 2018, thus highlighting the feasibility of de novo synthesis of smallpox [4]. Smallpox may re-emerge from deliberate or accidental release [5], and is a high-consequence event for which preparedness planning is needed [6]. Due to ageing, advances in medical therapies, transplantation and people living with immunosuppressive conditions such as HIV, the immunological status of the population has also changed dramatically since eradication of smallpox, with almost one in five people living with immunosuppression in Sydney, Australia [7]. A high proportion of people are unvaccinated, and vaccine-induced immunity in cohorts vaccinated before 1980 is waning [8–10]. In a modified SEIR deterministic model of smallpox transmission, we have shown that unprecedented rates of immunosuppression will result in increased morbidity and mortality of smallpox [7]. Planning for an epidemic of Planning for an epidemic of smallpox includes health system preparedness and resilience. In addition to vaccination, identifying and isolating cases to prevent further spread is influential in epidemic control [2].

The requirements for physical isolation space and surge capacity, as well as clinical health care workers (HCWs) to treat patients, must be considered in planning. In addition, an often-overlooked consideration is public health workers [11], who are required to conduct contact tracing and vaccination and monitor high and lower risk primary contacts to prevent secondary cases. Health systems capacity depends on both clinical acute care and public health personnel, hospital bed capacity, as well as protection of HCWs, who are often at high risk of nosocomial infections during serious epidemics [12]. Studies of smallpox re-emergence often assume health systems to be functioning and investigate only the role of vaccination on epidemic control [13–16]. There is a need to determine requirements for surge capacity within health systems in the event of re-emergent smallpox.

## Aims

To determine the capacity of the health system in Sydney, a city of 5.3 million people in Australia, during an epidemic of smallpox. Specifically, we aimed to determine hospital bedcapacity for isolation, public health workforce capacity for contact tracing and health care worker (HCW) personal protective equipment (PPE) requirements under different attack scenarios. We also aimed to test a worst case scenario among the range of possible attack scenarios and identify modifiable factors which would prevent a worst case scenario.

## Methods

We constructed a modified SEIR model for smallpox transmission based on a model published in our previous study [7]. Model parameters and their estimation have been previously described [7]. We assumed that the virus has not been genetically modified and that there is minimal residual immunity in the population from previous vaccination, as described in our previous study [7]. We assumed an initial attack size of 100, 1000 or 10,000 infected. Case isolation was assumed to reduce transmission to zero [2]. Given antivirals would be commenced after diagnosis and isolation, we assumed this effect would only apply in the healthcare setting and would not add to interruption of transmission above the effect of isolation alone, with the main transmission risk being in the community for undiagnosed or early cases prior to hospitalisation. We assumed that antivirals would therefore have no effect on community transmission, acknowledging that they would likely reduce morbidity and mortality for treated cases. We estimated number of hospital beds needed to control the epidemic, PPE requirements for clinical HCWs and public health workers required for contact tracing, under different scenarios.

### Mathematical model

We constructed a modified model for smallpox transmission. Population residual immunity and contact mixing are based on assumptions used in our previous study [7]. Using ordinary differential equations as described in S1 File, the population moves through the disease compartmental epidemiological states of being susceptible, exposed, infected and recovered (SEIR) from smallpox. Once infected, people move into the next state following disease duration rates. Euler’s approximation was used to estimate age-specific force of infection, assuming contact would be similar to observed patterns in the UK [17–19]. Different infectivity levels were based on the reproduction number for hemorrhagic, flat, ordinary and modified smallpox as previously described [7]. The force of infection was multiplied by a parameter (α1, α2, α3, α4) to account for different population susceptibility levels. Model parameters and their estimation are described in our previous study [7]. The model runs for 300 days. The model assumptions are shown in Table 1. To test the preparedness of the health system we assumed the base case response would be case isolation and treatment, contact tracing and ring vaccination. As the study was examining health system factors, ring vaccination was kept constant in the model at 90% with assumed adequate vaccine supply and trained vaccinators, and 95–98% vaccine efficacy for uninfected people and 50–53% for latent infected [2, 20–22] as described in S1 File.

**Table 1:** Model parameters and data sources.

Definition	Value	Source
Duration of quarantine for traced contacts	16.6 days	[2]
Duration of isolation for infectious contacts	25 days	[2]
Average number of contacts per case	11	[19]
Proportion of contacts traced around an infected case	90%, Sensitivity analysis with 70% and 50%	[2]
[Proportion of cases that get isolated once infected and symptomatic	95%, Sensitivity analysis on with 70% and 50%	[2]
Time of starting intervention	At day 15, 20 and 30 after release, corresponding to 3, 8 and 18 days after the onset of symptoms of the index case, using an average incubation period of 12 days.	[2]
Initial infected	100, 1000, 10000	

#### Hospital bed requirements

We estimate the number of hospital beds in Sydney using data published for 2015–2016 in NSW [18]. In NSW for 2015–2016 there were 21152 beds available from public hospitals (2.78 per 1000 population) and 8184 beds available from private hospitals (1.07 per 1000 population). This was resized for the Sydney population. The number of hospital beds needed for case isolation was then modelled under different scenarios based on variation of response time (T), the percentage of infected cases isolated each day and how many contacts were traced. We tested if the number of available hospital beds in Sydney will be enough to isolate up to 95% of all new infected cases every day, under different scenarios.

#### Clinical health workforce and PPE requirements

The clinical health workforce was estimated by the number of HCWs in Sydney for 2016/2017, including Aboriginal and TSI health practitioners, Chinese medicine practitioners, dental practitioners, medical practitioners, radiation therapists, nurses and midwives, occupational therapists, pharmacists and ambulance services workers [23]. The total estimated health workforce number was 147997 for NSW, with a total population of 7.7 million in 2016 [24]. We applied the same percentage adjusted for the Sydney population from the same year, 5.25 million [25]. HCW distribution by age group was estimated using national and global health worker data [26, 27]. We estimated, based on epidemic size and duration, the amount of respiratory PPE (N95 respirators) required for Sydney clinical HCWs assuming two respirators per shift per HCW. This is based on recommendations that disposable respirators should not be re-used, and the fact that a standard shift for a HCW would include at least one break, after which a new respirator would need to be used.

#### Public health workforce for epidemic control

There are no published data to estimate the public health workforce, comprising trained public health officers working in health departments and capable of conducting contact tracing and outbreak investigation, as public health workers are not registered health practitioners. The only uniform qualification in public health is a Master of Public Health (MPH) and similar degrees. Whilst there are a large number of MPH graduates in Australia, the number working in government public health roles would be a minority. It should also be noted that a MPH does not equip people with the skills for field response to an epidemic. There are approximately 300 alumni of the national Field Epidemiology Training Program (FETP). In addition, there is a medical specialisation in public health medicine for a relatively small number of medical doctors, with an estimated 244 full time equivalent public health physicians nationwide in 2017 [17]. Based on discussions with national experts we estimated there are approximately 1000 skilled public health officers in Australia, although the actual number may be lower. The public health workforce was calculated using estimates of MPH graduates currently working in government, FETP graduates or current FETP trainees and public health physicians. An optimistic assumption of 1500 public health officers nationally was used to estimate the number working in Sydney.

#### Contact tracing requirements

In the base case, we assumed 90% of contacts would be traced and 95% infectious people would be isolated. We used age specific contacts rates, with an average of 11 contacts per case based on European social mixing data [19]. We estimated the number of public health workers required to conduct contact tracing under different scenarios. Given contact tracing may require complex communications and travel over large geographic distances, we assumed one public health officer could trace 10 contacts per day. Australian guidelines for management of smallpox state that isolation is needed for non-immune Category A (high risk) contacts, in individual rooms with supervision by vaccinated staff [28]. We conservatively assumed at least 50% of contacts traced would be Category A, and would require supervised quarantine. Data from tuberculosis studies [29] as well as estimated social contact matrices suggest one person [19], on average, has 9–11 contacts at reasonable risk of infection. The closest of these contacts would include household contacts (about 3–4 people), plus 1–2 others in work or friendship circles. This would be about half of the 9–11 contacts. The number of contacts needed to be traced and managed was estimated based on attack size, time to response (T) and the percentage of infected cases isolated each day. A contact tracing day was defined as one day entirely spent tracing contacts per public health worker.

#### Sensitivity analysis

A sensitivity analysis was conducted on attack size, the proportion of infectious cases isolated and contacts traced, as well as time to commencing the response. To illustrate the difference in epidemic size between a single index case of smallpox imported from overseas, compared to a primary attack which results in 100 or 1000 simultaneous first-generation cases, we modelled the epidemic resulting from 1, 100 or 1000 initial first generation cases. The size of an attack is unknown, and would depend on the technical sophistication of aerosol dispersion of variola. To account for this uncertainty, we explored the influence of attack scenarios of 100, 1000 and 10000 initial infected as a wide range of possible attack sizes, to determine the impact of attack size on epidemic control.

Delays in diagnosis and time to obtaining laboratory confirmation could vary the time of onset of the response. We therefore varied the time of the response commencing between T = 15, 20 and T = 30 days following virus release. Given an average incubation period of 12 days for smallpox [2], this corresponds to day 3, 8 and 18 after the onset of symptoms of the index case.

## Results

We estimated 14595 public hospital beds and 5618 private hospital beds in Sydney. We estimated there are 100638 clinical HCWs in Sydney, the majority (65%) aged 30–49 years old, 51% nurses and 18% doctors [27]. We estimated a public health workforce of 1500 nationally, with approximately 330 public health workers in Sydney.

**Fig 1:**
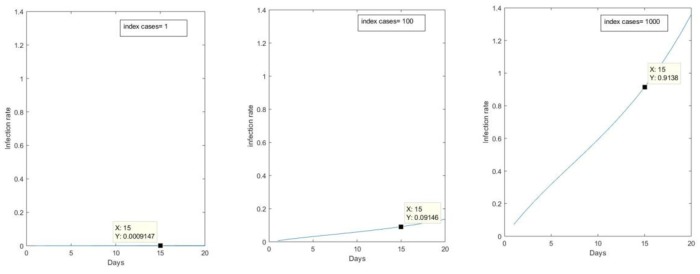
Comparison of epidemic size resulting from a single imported case and a deliberate release scenario with 100 and 1000 initial infected cases.

Fig 1 shows the relative epidemic size of a deliberate release scenario with 100 or 1000 initial infected compared to a single importation of smallpox from an epidemic overseas, to illustrate the potential scale of the required public health response.

**Fig 2:**
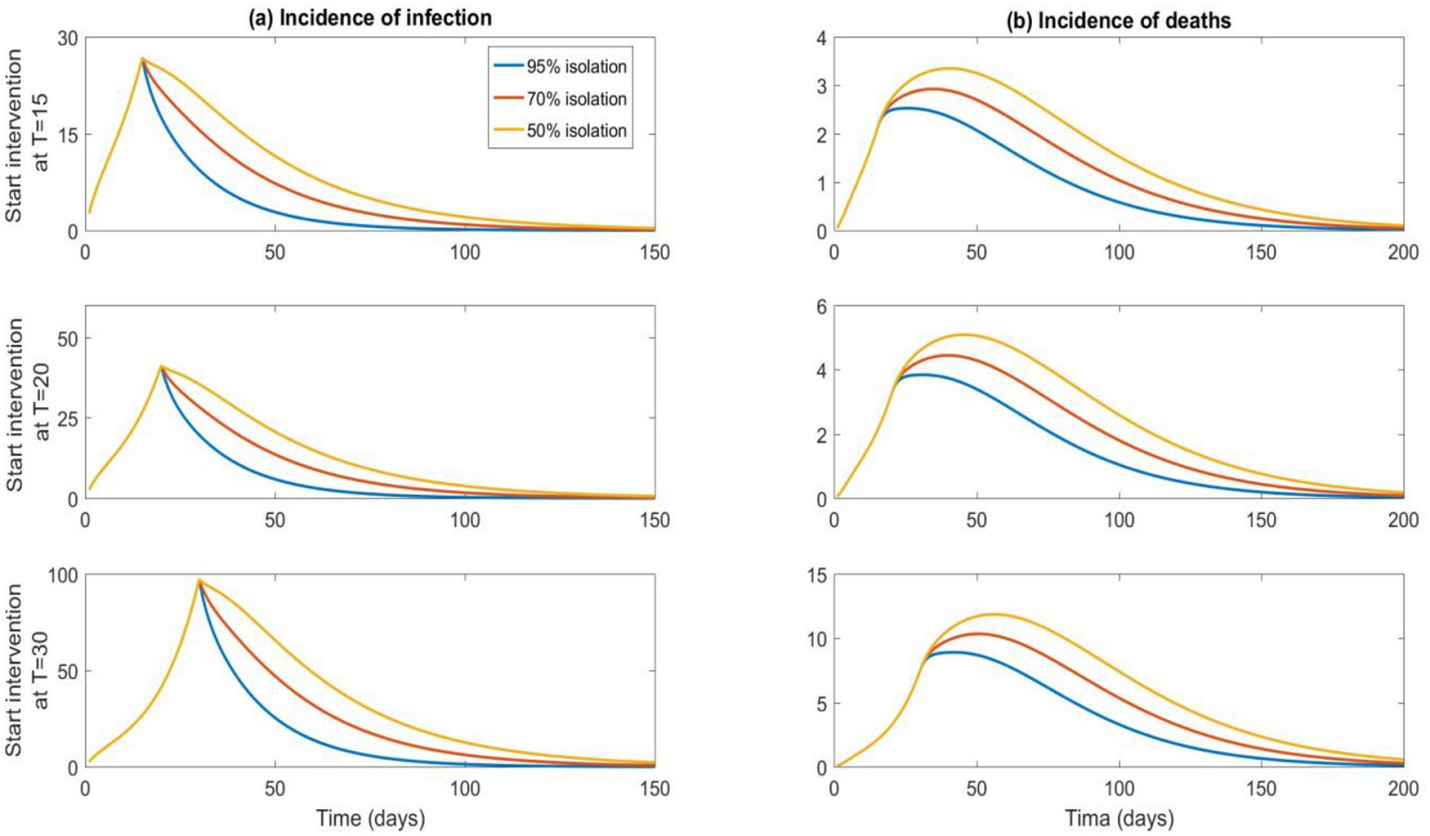
Incidence of infection and death: Sensitivity analysis on proportion of infected cases isolated and time from virus release to starting interventions. Results shown for 100 initial infected and 90% contacts traced/vaccinated.

The higher the initial number infected, the more rapid and severe the epidemic. Without intervention, the death rate will reach an incidence (number of new infected people per day) rate of 8 deaths per 1000 population per day. The overall reproductive number was estimated to be 4.6. Fig 2 shows the influence on infections and deaths by varying time to response and case isolation rates. Both timing and isolation rates of infected cases are highly influential in outbreak control. With 100 infected initially, when isolation decreases, the deaths increase from a maximum of 2, 4 and 9 per day in the best scenario with 95% isolation (for T = 15,20 and 30 respectively), to 3.5, 5.5 and 12 per day with only 50% of cases isolated (Fig 2).

**Fig 3:**
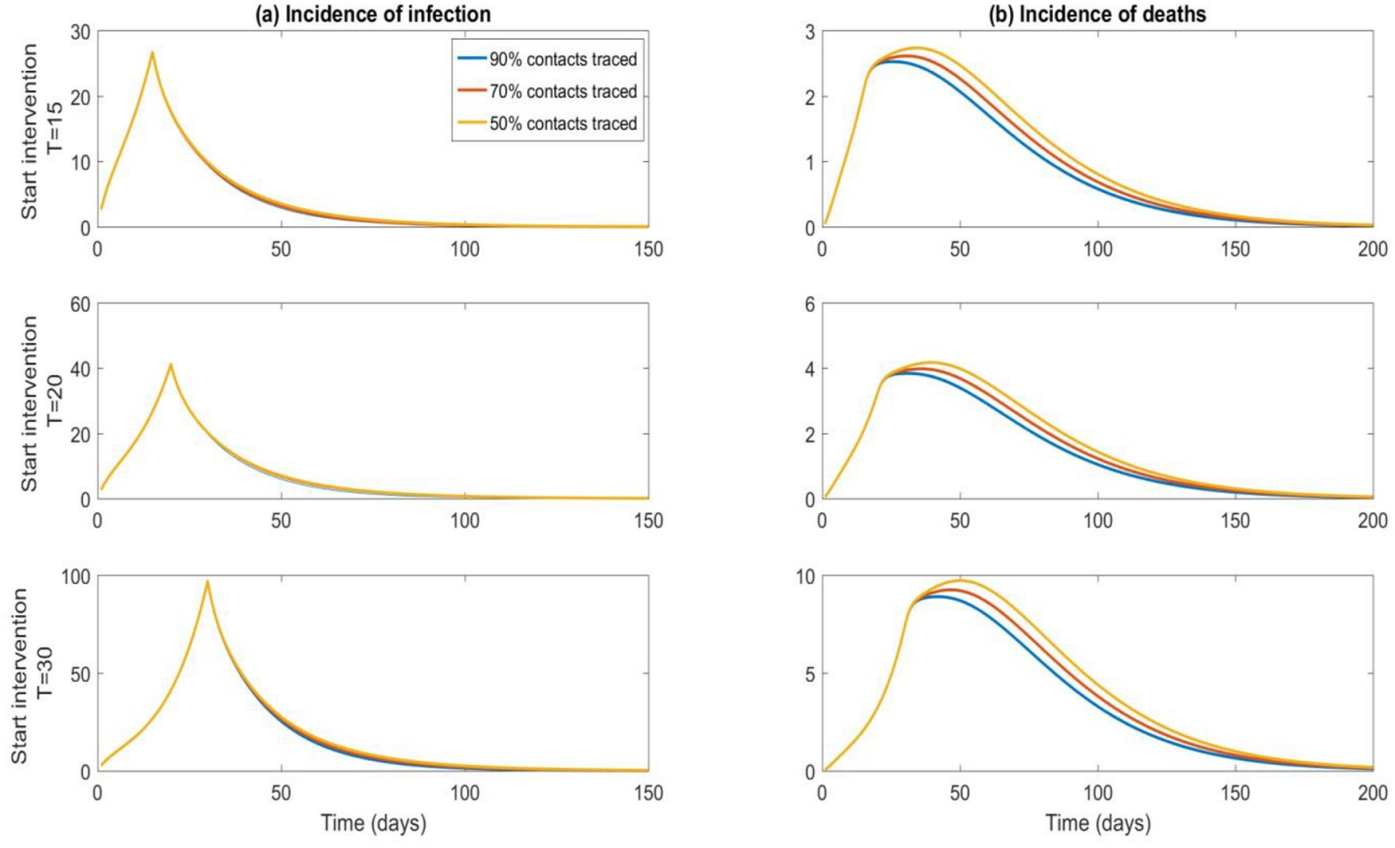
Incidence of infection and death: Sensitivity analysis on proportion of contacts traced and time from virus release to starting interventions. Results shown for 100 initial infected and 95% infectious cases isolated.

Fig 3 shows the influence of varying time of starting the intervention and varying percentage of contacts traced on the incidence of infections and deaths with case isolation constant at 95%. With a high proportion of cases isolated, outbreak control can be achieved within 100 days even with only 50% of contacts traced.

**Fig 4:**
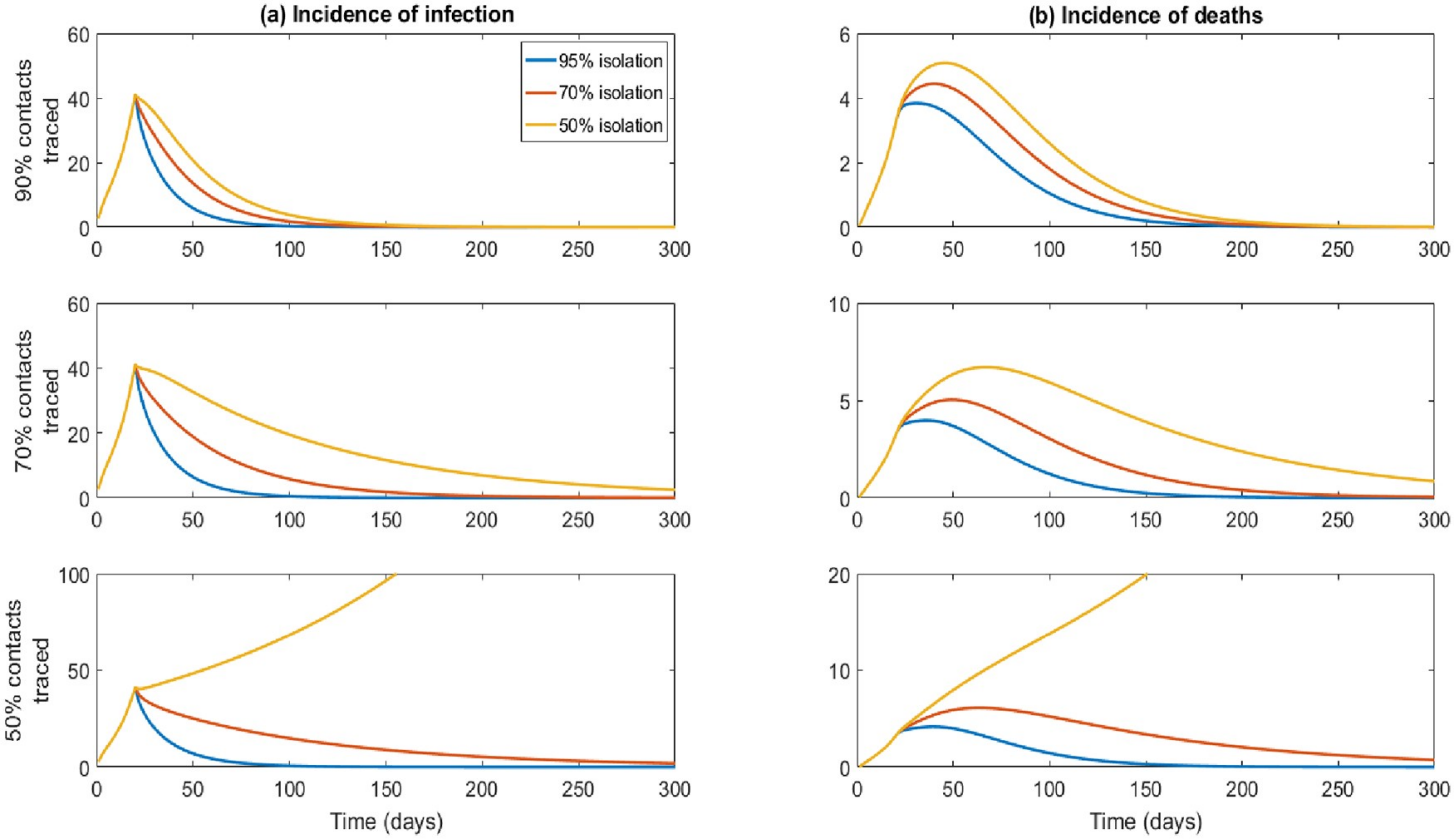
Incidence of infection and death: Sensitivity analysis on proportion of cases isolated and contacts traced. Results shown for 100 initial infected and T = 20 starting intervention.

Fig 4 shows the effect of varying both case isolation and contact tracing rates, with the intervention commencing at 20 days—case isolation is more influential, with epidemic control severely impacted when isolation and contact tracing falls to 50%.

In Table 2 we show the impact of the epidemic (total cases and contacts needed to be traced) by varying case isolation rates. The total number of cases range from 528 to 285,400; and contacts that need to be traced between 1277 and 940350 in the best- and worst-case scenarios respectively.

**Table 2:** Total number of infected cases and contacts needed to be traced and quarantined by the end of the epidemic with varying case isolation rates and 90% contacts traced.

Initial contacts infected		100			1000			10000		
Day to start of response	Time post-release	T = 15	T = 20	T = 30	T = 15	T = 20	T = 30	T = 15	T = 20	T = 30
	Time from symptom onset	T = 3	T = 8	T = 18	T = 3	T = 8	T = 18	T = 3	T = 8	T = 18
95% of cases isolated	Contacts	1277	1974	4655	12751	19677	46199	125190	191040	429340
	Cases	528	819	1949	5275	8181	19409	52231	80486	186278
70% of cases isolated	Contacts	2187	3361	7920	21803	33443	78247	211420	318580	696490
	Cases	704	1089	2582	7031	10850	26621	68968	105235	238097
50% of cases isolated	Contacts	3122	4787	11278	31075	47524	110790	296940	442810	940350
	Cases	886	1365	3232	8828	13578	31919	85551	129323	285400

### Number of hospital beds needed for case isolation

**Fig 5:**
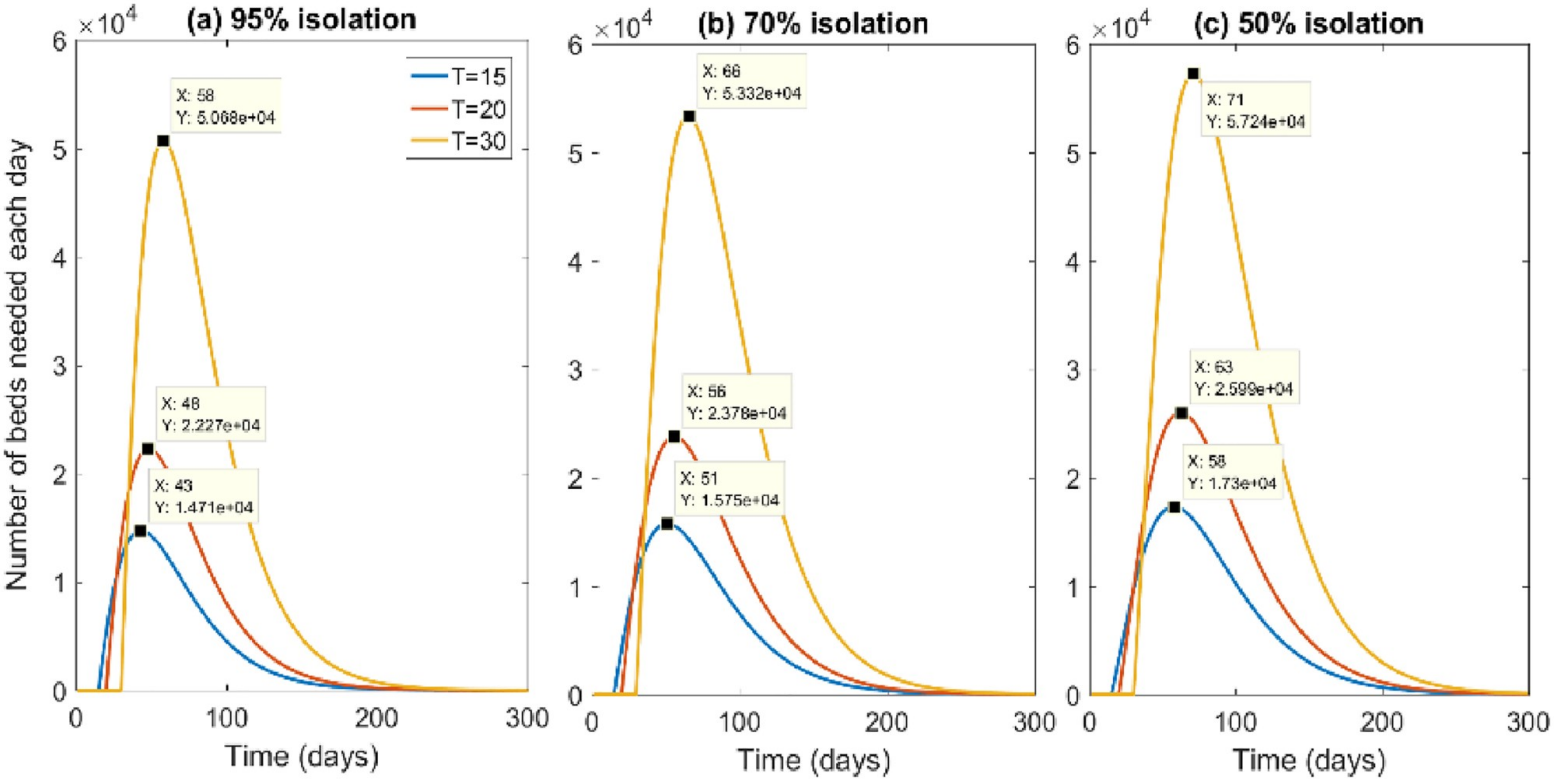
Total number of beds needed each day of the outbreak by time of starting intervention and percentage of case isolation with 10000 initially infected.

We estimated 14595 public hospital beds and 5618 private hospital beds in Sydney. The modelled maximum number of people isolated at the same time is about 2.3 and 5.5 times the initial number of infected if we start intervention respectively at T = 20 and at T = 30 and peaks 28 days after the response commences. Therefore, if the initial number of infected is 100 or 1000, the available beds will not be completely exhausted, but treatment capacity for other illnesses may be impacted. In the case of 100 initial infected, the maximum beds usage will reach 1.6% and 3.8% of Sydney public available beds, if the response starts at time T = 20 and T = 30 days from the virus release respectively. If the initial number of infected is 1000, the maximum beds usage will reach 16.1% and 37.9% of Sydney public available beds 28 days after the response commences, if the response starts at time T = 20 and T = 30 days from the virus release respectively. However, in the case of 10000 initial infected, the available hospital beds will be all used in the first few days of the response. Fig 5 shows the hospital bed usage in the worst-case scenario of 10000 initially infected, with varying start times of the response.

Maximum number of beds needed at the same time and day shown in the square windows. With 10000 initial infected if we start the intervention at T = 20 from virus release, 1703 beds will be used the first day, 3356 the second day. At 7 and 13 days after commencing the intervention (at day 27 and 33) more than 50% and 80% of the total beds in Sydney hospitals will be needed. At day 39 post-attack, 19 days after starting the intervention, 100% of all public and private beds will be used. If the intervention is delayed to day T = 30, almost 50% of the available beds will be used in the first 2 days, 80% at day 5 of response will be used and at day 6 of the response (T = 36 after the attack) all available public and private beds will be used. Table 3 shows the time to occupancy of all available hospital beds at levels of 20% or greater. Whilst an attack of 100 initial infections does not reach 20% of beds under any scenario, in the worst-case scenario (response commencing at day 30) 100% of beds will be used by day 36.

**Table 3:** Time (days) to when 20% or more of available hospitals beds are used for smallpox cases, by intervention starting time (T) and number of initial infected. Results showed for 95% of new infected isolated.

Percenta-ges of beds used	20%			50%			80%			100%			Maximum number of beds used in the same day (% of the total)		
Start of response (days)	T = 15	T = 20	T = 30	T = 15	T = 20	T = 30	T = 15	T = 20	T = 30	T = 15	T = 20	T = 30	T = 15	T = 20	T = 30
10000 initial infected	19	23	31	27	27	33	NR	33	35	NR	39	36	14710(73%)	22270	50680
1000 initial infected	NR	NR	44	NR	NR	NR	NR	NR	NR	NR	NR	NR	1487 (7%)	2264 (11%)	5291 (26%)
100 Initial infected	NR	NR	NR	NR	NR	NR	NR	NR	NR	NR	NR	NR	149 (less than 1%)	227 (1%)	531 (3%)

NR = never reaches 20% of available beds

### Clinical HCW and PPE requirements

The number of HCWs required and the PPE they need will be proportionate to the number of cases requiring treatment (Table 2). In scenarios described above where cases (beds) exceed 100% of available beds, staffing requirements will increase 100%, unless reduced staff/patient ratios are implemented.

Estimating a minimum of 2 disposable respirators a day per HCWs for 150 days, over 30 million respirators will need to be stockpiled for all 100638 clinical HCWs in Sydney. This number can be used to estimate requirements based on the estimated percentage of the clinical workforce needed for the epidemic, which will be proportionate to the number of cases requiring treatment (Table 2). If 10% of clinical HCWs are involved in care of smallpox patients, over 3 million respirators will be needed. If the epidemic is not controlled within 300 days this number will be doubled.

### Contact tracing requirements

The public health staff (PHS) required to conduct contact tracing will depend on how many contacts one person can trace per day and the number of available PHS, estimated to be 330 in Sydney. If one PHS can trace 10 contacts a day, then in the best-case scenario of 1277 contacts, over 127 contact tracing days are required, based on the number of contacts in Table 2 above. In the worst-case scenario, 940,350 contacts and 94,035 contact tracing day are required. In the worst-case scenario, 330 PHS would work over 285 days each doing contact tracing. If half of contacts are high-risk, quarantine spaces will be required for 638 to 470,175 contacts.

## Discussion

In the case of a smallpox release in Sydney, a high-income, well-resourced city of over 5.3 million people, health system impacts may be substantial under some scenarios as shown in our model. We showed if smallpox arises overseas and is imported as a single case into Australia by travel, control will be far easier than under an attack scenario. We showed that influential factors on epidemic impact are the size of the initial attack, time to commencing the response, case isolation rates and contact tracing for ring vaccination. Whilst both are influential, case isolation is more influential than contact tracing. These public health interventions depend on physical and human resources, including clinical and public health workforce. Whilst the size of an attack may not be within our control, other the influential factors are modifiable and potentially within our control. If the initial attack size is 100–1000 and the response is rapid, an outbreak of smallpox can be controlled with case isolation, contact tracing and vaccination. However, if the response is delayed to 30 days or longer (which equates to about 2 weeks after the first symptoms occur), or if the attack infects 10000 people, epidemic control will be much more challenging, and the health systems impacts will be substantial. In the worst-case scenario, available hospital beds will be exceeded in less than 40 days. The requirement for hospital beds for isolation of cases will vary from up to 4% to 100% of all available beds depending on the size of initial release and speed of response. Even in the mid-range scenario of 1000 initial cases, up to 40% of all available hospital beds will be required for smallpox control. This does not account for the facilities required for quarantine of contacts, which must additionally be planned for, and in the worst-case scenario would require over 400,000 high risk contacts to be quarantined. Quarantine and isolation capacity are critical to epidemic control. Planning for surge bed capacity using available guidelines should be undertaken [30], and back up plans such as the use of community halls, school buildings, hotels or other large buildings should be made to ensure that that other viable isolation sites are pre-designated as smallpox treatment centres and available. During the 2009 pandemic of influenza, which was reportedly not as severe as expected, studies reported a tripling of patient presentations to hospital [31]. Plans for managing hospital bed capacity in the event of a large initial attack should also be made, including designation of specific treatment facilities, cancellation of elective surgery and decanting of patients with non-urgent other conditions into private hospitals or other facilities. The capacity for hospital beds for non-smallpox patients who require urgent hospitalisation must also be considered, and in some scenarios, the care of patients with urgent non-infectious conditions such myocardial infarction or stroke, may be compromised by lack of hospital capacity and staffing shortages.

Rapid response time is critical and becomes even more critical when the initial infected number is higher. Responding more than 20 days from the virus release (which means commencing the public health response within 7 days of symptom onset, given an average 12 day incubation period) will result in a more severe outbreak. Whether it is feasible to commence response within the best-case scenario of 15 days post-release (or 3 days after symptom onset) is unknown, but unlikely. A rapid response depends on very early detection and diagnosis, as well as prompt commencement of case finding, isolation, contact tracing and vaccination. Practically, the target for reducing the time to response is in early diagnosis, which depends on awareness first, followed by diagnostic test.

Delays may occur if the diagnosis is missed in the index case. Recent examples of serious emerging infectious diseases where the diagnosis was missed include Ebola in Nigeria and the US, both of which occurred during the height of the West African epidemic when media reports were at a peak and awareness should have been high [32, 33]. The largest epidemic of MERS Coronavirus outside the Arabian peninsula occurred in South Korea following a missed diagnosis and failure of triage in a patient with a relevant travel history and a respiratory clinical syndrome [34]. The last European epidemic of smallpox in 1972 also involved a missed diagnosis, when a traveller to the Middle East returned to Yugoslavia, which had been free of smallpox for 30 years. The patient had haemorrhagic smallpox, which was misdiagnosed as a severe adverse reaction to an antibiotic, and smallpox was not suspected until second generation cases began occurring, resulting in an outbreak of 175 cases [35]. Excellent surveillance systems and triage protocols for early detection of low probability, high impact outbreaks such as smallpox is recommended. Improving diagnosis requires triage protocols and rapid diagnostics, the latter being useful only if the diagnosis is suspected clinically in the first instance. Other avoidable delays in response could include having pre-vaccinated first responder teams, pre-designated isolation and quarantine facilities, and rapid human resources and surge capacity scale up plans [36].

We have showed that epidemic control is highly sensitive to case isolation rates, which need to be maintained at high levels. Identifying and isolating less than half the new infected cases and tracing only half of all contacts will result in a blow-out of the epidemic. Space and human resource requirements for case finding, isolation, contact tracing, vaccination and quarantine are therefore essential for preparedness planning. Physical space requirements extend beyond isolation of smallpox cases, to quarantine of contacts. In the worst-case scenario, almost one million contacts need to be traced and there will be a lack of physical space for quarantine of high-risk contacts. Plans for home quarantine and surveillance of contacts should also be undertaken and will require adequately trained personnel. The speed and effectiveness of contact tracing is also critical to the success of ring vaccination and will require an adequately trained critical mass of public health workers and epidemiologists, separate from the clinical workforce. In Australia, as a federation, this will rely on State and Territory capacity, and cross-border mobilisation of jurisdictional capacity in the event of a smallpox epidemic, and estimation of the current and required capacity for such an event. The fact that public health personnel are not registered as health practitioners or documented in any other centralised way, makes it more challenging to rapidly mobilise suitably qualified and experienced personnel for a large-scale epidemic response. This is a policy consideration that could be addressed as part of pandemic and health emergency planning, which may strengthen response. Contact tracing may need to rely on community volunteers, as the available public health workforce will be inadequate in a large epidemic.

Staff surge requirements would track parallel to bed requirements and would be over 100% in some scenarios. The need for a clinical health workforce to treat smallpox will be high, with case numbers in the 1000s to 100,000s in many of the scenarios modelled, and just over 100,000 clinical HCWs in Sydney. Limiting the number of HCWs working in designated smallpox facilities is a sensible strategy. A possible approach to such a scenario would be a reduction of staff to patient ratios, as well as using trainee HCWs. Protection of these clinicians is key, with vaccination being the mainstay. Up to 100,000 doses of vaccine will need to be reserved for clinical HCWs and plans in place to commence vaccination. PPE will not be an alternative to vaccination, but an additional protective measure for HCWs. Today, work health and safety requirements would dictate that PAPRs or disposable respirators with a hood and coveralls be available to clinicians treating smallpox cases. Perceived lack of protection during a serious emerging infection outbreak may result in refusal to work or industrial action by HCWs [37]. HCW may be well protected by PPE, but there is large uncertainty around effectiveness of PPE. Studies of other viruses transmitted by the respiratory route suggests good effectiveness of respirators against smallpox [38]. It should be noted that surgical masks are unlikely to offer protection to HCWs based on available data [38]. Stockpiling may provide a short duration of supplies. The modelled epidemic may run for 150–300 days or more, depending on the scenario. A very large quantity of respirators may need to be stockpiled, depending on the percentage of HCW involved in direct care of smallpox patients. Given the likely duration of an epidemic, plans should put in place for rapid procurement of PPE supplies beyond the stockpiled capacity. Strategies to minimise the number of HCW treating each case of smallpox, including using designated smallpox hospitals, will reduce the quantity of PPE required.

Early identification of the epidemic, high rates of case isolation, excellent contact tracing and vaccination, and protection of HCWs are the key influential components of epidemic control. Failure in any of these could severely compromise the capacity of the health system. Australia has a detailed plan for smallpox response,[39] and we have outlined key influential parameters for disease control which can add further guidance on mitigating severe outcomes in both the planning and response stages. Excellent surveillance systems and triage protocols for early detection of low probability, high impact outbreaks such as smallpox can make a difference, given the criticality of timing of the response and better prospects of epidemic control in the early stages. Planning for the health system should consider rapid surge capacity for beds, strategies to create and staff make-shift designated smallpox treatment facilities, and protection of HCWs at all levels of care. Requirements for contact tracing are substantial and may require mobilisation of community volunteers and additional space for quarantine and surveillance of high-risk contacts. Designated surge smallpox facilities and plans for management of other urgent health conditions should be considered. We have outlined several modifiable factors which, with good planning, can ensure adequate health system capacity in the event of a smallpox epidemic.

## Supporting information

S1 File Supplementary information on the model. (DOCX)
