# Supplementary material for: Health system capacity in Sydney, Australia in the event of a biological attack with smallpox
Source: PLoS One. 2019 Jun 14;14(6):e0217704. doi: 10.1371/journal.pone.0217704 (PMC6568391; doi:10.1371/journal.pone.0217704)
Supplement: S1 File — (DOCX) [file pone.0217704.s001.docx]

**Force of infection**

The age-specific rates at which a susceptible person becomes infected ($\lambda$) is a combination of β (probability of becoming infected per contact), c (the number of contacts per unit time) and the infectious prevalence. To include age-dependency, we used Euler’s discretization of the continuous variable ‘age’, so that force of infection is represented by

$$\lambda=\sum_{j=1}^{6} \frac{\sum_{i=1}^{15} \beta_{j}*c_{i,i}*I_{i,j}}{N}$$

While, $\lambda_{2}$ represents all the rest of the contacts traced but not infected

$$\lambda_{2}=\sum_{j=1}^{6} \frac{\sum_{i=1}^{15} {(1-\beta}_{j})*c_{i,i}*I_{i,j}}{N}$$

Where j=1,…,6 represents the 6 infectious levels, (haemorrhagic, flat, ordinary, modified, ordinary and modified with reduced contacts) and i=1,…,15 represents the age groups within the contact matrix.

**Model’s differential equations**

A previous model structure (1) was revisited and used.

SUSCEPTIBLES

$dS1=-\alpha_{1}*\lambda*S1-\rho*\lambda_{2}*S1+(1-{ve}_{2})*C1i/q_{1}$; sever immunocompromised

$dS2=-\alpha_{2}*\lambda*S2-\rho*\lambda_{2}*S2+(1-{ve}_{2})*C2i/q_{1};$ mild immunocompromised

dS3$=-\alpha_{3}*\lambda*S3-\rho*\lambda_{2}*S3+(1-{ve}_{2})*C3i/q_{1}$; healthy never vaccinated

dS4$=-\alpha_{4}*\alpha_{3}*\lambda*S4-\rho*\lambda_{2}*S4+(1-v_{2})*C4i/q_{1}$; healthy previously vaccinated

dS5$=-\alpha_{5}*\lambda*S5-\rho*\lambda_{2}*S5+(1-{ve}_{2})*C5i/q_{1}$; HCWs never vaccinated,

dS6$=-\alpha_{4}*\alpha_{5}*\lambda*S6-\rho*\lambda_{2}*S6+(1-v_{2})*C6i/q_{1}$; HCWs previously vaccinated

LATENT UNTRACED

$$dE1n=(1-\rho)* \alpha_{1}*\lambda*S1-E1n/d_{1};$$

$$dE2n=(1- \rho)* \alpha_{2}* \lambda*S2-E2n/d_{1};$$

$$dE3n=(1- \rho)* \alpha_{3}* \lambda*S3-E3n/d_{1};$$

$$dE4n=(1- \rho)* \alpha_{4}*\alpha_{3}* \lambda*S4-E4n/d_{1};$$

$$dE5n=(1- \rho)* \alpha_{5}* \lambda*S5-E5n/d_{1};$$

$$dE6n=(1- \rho)* \alpha_{4}* \alpha_{5}* \lambda*S6-E6n/d_{1};$$

LATENT TRACED which we will vaccinate all of them and keep them isolated

$$dE1i=\rho* \alpha1*\lambda*S1-(1-{ve}_{1})*E1i/d_{1}-{ve}_{1}*E1i/q_{1};$$

$$dE2i=\rho* \alpha_{2}* \lambda*S2-(1-{ve}_{1})*E2i/d_{1}-{ve}_{1}*E2i/q_{1};$$

$$dE3i=\rho*\alpha_{3}* \lambda*S3-(1-{ve}_{1})*E3i/d_{1}-{ve}_{1}*E3i/q_{1};$$

$$dE4i=\rho* \alpha_{4}*\alpha_{3}* \lambda*S4-(1-{ve}_{1})*E4i/d_{1}-v_{1}*E4i/q_{1};$$

$$dE5i=\rho* \alpha_{5}* \lambda*S5-(1-{ve}_{1})*E5i/d_{1}-{ve}_{1}*E5i/q_{1};$$

$$dE6i=\rho* \alpha_{4}* \alpha_{5}* \lambda*S6-(1-{ve}_{1})*E6i/d_{1}-v_{1}*E6i/q_{1};$$

UNINFECTED CONTACT TRACED

$$dC1i=\rho*\lambda_{2}*S1-C1i/q_{1};$$

$$dC2i=\rho*\lambda_{2}*S2-C2i/q_{1};$$

$$dC3i=\rho*\lambda_{2}*S3-C3i/q_{1};$$

$$dC4i=\rho*\lambda_{2}*S4-C4i/q_{1};$$

$$dC5i=\rho*\lambda_{2}*S5-C5i/q_{1};$$

$$dC6i=\rho*\lambda_{2}*S6-C6i/q_{1};$$

ISOLATION

$$dQ=\theta*(E1n/d_{1}+E2n/d_{1}+E3n/d_{1}+E4n/d_{1}+E5n/d_{1}+E6n/d_{1})+$$

$$(1-{ve}_{1})*(E1i/d_{1}+E2i/d_{1}+E3i/d_{1}+E4i/d_{1}+E5i/d_{1}+E6i/d_{1})-Q/q_{2};$$

INFECTIOUS with the 4 different diseases types

$$dI1=(1-\theta)*(E1n/d_{1}+2*m_{1}*E2n/d_{1}+m_{1}*(E3n/d_{1}+E5n/d_{1})+$$

$$\alpha_{4}*m_{1}*(E4n/d_{1}+E6n/d_{1}))-I1/dz; haemorraghic$$

$$dI2=(1-\theta)*(2*m_{2}*E2n/d_{1}+m_{2}*(E3n/d_{1}+E5n/d_{1})+$$

$$\alpha_{4} *m_{2}*(E4n/d_{1}+E6n/d_{1}))-I2/dz; flat$$

$$dI3=(1-\theta)*((1-2*(m_{1}+m_{2}))*E2n/d_{1}+(1-(m_{1}+m_{2}))*$$

$$(E3n/d_{1}+E5n/d_{1})+(1-\alpha_{4}*(m_{1}+m_{2})- ɣ)*(E4n./d_{1}+E6n./d_{1}))-I3./{do}_{1}; ordinary$$

$$dI4=(1-\theta)*( \alpha_{4}*(E4n/d_{1}+E6n/d_{1}))-I4/{dm}_{1}; modified$$

$$dI5=I3/{do}_{1}-I5/{do}_{2}; ordinary reduced contacts$$

$$dI6=I4/{dm}_{1}-I6/{dm}_{2}; modified reduced contacts$$

RECOVERED

$dR=(1-\mu_{3})*I1/dz+(1-\mu_{2})*I2/dz+(1-\mu_{1})*I5/{do}_{2}+I6/{dm}_{2}+0.7*Q/q_{2};$

DEATHS

$$dD=\mu_{3}*I1/dz+\mu_{2}*I2/dz+\mu_{1}*I5/{do}_{2}+0.3*Q/q_{2};$$

SUCCESSFULLY VACCINATED

$dV={ve}_{2}*(C1i+C2i+C3i+C5i)/q_{1}+v_{2}*(C4i+C6i)/q_{1}+{ve}_{1}*E1i/q_{1}+{ve}_{1}* E2i/q_{1}+{ve}_{1}*E3i/q_{1}+v_{1}*E4i/q_{1}+{ve}_{1}*E5i/q_{1}+v_{1}*E6i/q_{1};$

Parameter estimation for smallpox transmission, duration stages and age distribution rates for haemorrhagic, flat, ordinary and vaccine modified smallpox are listed in the technical appendix from our previous study (2), while parameters involved in the implementation of ring vaccination in the model and additional parameters are listed in the following table

| Symbol | Definition | Value | Source |
| --- | --- | --- | --- |
| $\alpha_{5}$ | Susceptibility of HCWs | 3 |  |
| $m_{1}$ | Age specific distribution rate of haemorrhagic smallpox in healthy unvaccinated people | See Technical Appendix Table 3 | (2) |
| $m_{2}$ | Age specific distribution rate of flat smallpox in healthy unvaccinated people | See Technical Appendix Table 3 | (2) |
| $\theta$ | Percentage of infectious people isolated | 90%  Sensitivity analysis with 70% and 50% | (3) |
| $\rho$ | Percentages of contacts traced and vaccinated | 95%  Sensitivity analysis on with 70% and 50% | (3) |
| $q_{1}$ | Duration of isolation for contacts traced after vaccination | 17 | (1) |
| $q_{2}$ | Duration of isolation for infectious symptomatic cases | 25 | (1) |
| ${do}_{1}$ | Infectious duration for ordinary smallpox with full R0 | 2 days |  |
| ${do}_{2}$ | Infectious duration for ordinary smallpox with reduced R0 | 14 days |  |
| ${dm}_{1}$ | Infectious duration for modified smallpox with full R0 | 3 days |  |
| ${dm}_{2}$ | Infectious duration for modified smallpox with reduced R0 | 13 days |  |
| $dz$ | Infectious duration for haemorrhagic and flat smallpox with reduced R0 from day 1 | 16 days |  |
| $v_{1}$ | Vaccine effectiveness in latent infected previously vaccinated | 0.53 | (4) |
| $v_{2}$ | Vaccine effectiveness in uninfected previously vaccinated | 0.98 | (4) |
| ${ve}_{1}$ | Vaccine effectiveness in latent infected never vaccinated before | 0.50 | (5,6) |
| ${ve}_{2}$ | Vaccine effectiveness in uninfected never vaccinated before | 0.95 | (7) |

**References**

1. Gani R, Leach S. Transmission potential of smallpox in contemporary populations. Nature [Internet]. 2001 Dec 13 [cited 2017 Aug 11];414(6865):748–51. Available from: http://www.nature.com/doifinder/10.1038/414748a

2. MacIntyre CR, Costantino V, Chen X, Segelov E, Chughtai AA, Kelleher A, et al. Influence of Population Immunosuppression and Past Vaccination on Smallpox Reemergence. Emerg Infect Dis [Internet]. 2018 Apr [cited 2018 Mar 20];24(4):646–53. Available from: http://wwwnc.cdc.gov/eid/article/24/4/17-1233_article.htm

3. Fenner F, Henderson D, Arita I, Ježek Z LI. Smallpox and its eradication.

4. Massoudi MS, Barker L, Schwartz B. Effectiveness of Postexposure Vaccination for the Prevention of Smallpox: Results of a Delphi Analysis. J Infect Dis [Internet]. 2003 Oct 1 [cited 2018 Jan 8];188(7):973–6. Available from: https://academic.oup.com/jid/article-lookup/doi/10.1086/378357

5. Nalca A, Zumbrun EE. ACAM2000: the new smallpox vaccine for United States Strategic National Stockpile. Drug Des Devel Ther [Internet]. 2010 May 25 [cited 2017 Aug 11];4:71–9. Available from: http://www.ncbi.nlm.nih.gov/pubmed/20531961

6. Sato H. Countermeasures and vaccination against terrorism using smallpox: pre-event and post-event smallpox vaccination and its contraindications. Environ Health Prev Med [Internet]. 2011 Sep [cited 2018 Sep 7];16(5):281–9. Available from: http://www.ncbi.nlm.nih.gov/pubmed/21431786

7. Mack TM. Smallpox in Europe, 1950-1971. J Infect Dis [Internet]. 1972 Feb [cited 2018 Mar 20];125(2):161–9. Available from: http://www.ncbi.nlm.nih.gov/pubmed/5007552
